# Habit Learning in OCD: Preliminary Data from a Spanish Sample

**DOI:** 10.1192/j.eurpsy.2024.734

**Published:** 2024-08-27

**Authors:** M. Prime Tous, C. Lopez Solà, L. Hermida, M. A. Fullana

**Affiliations:** ^1^Child and Adolescent Psychiatry and Psychology Department, Institute of Neurosciences; ^2^Adult Psychiatry and Psychology Department, Institute of Neurosciences, Hospital Clínic, Barcelona, Spain

## Abstract

**Introduction:**

Instrumental learning involves goal-directed and habitual systems. The Slips-of-Action Task (SOAT) is extensively used to measure habit tendencies and the likelihood of making erroneous responses for devalued outcomes. The SOAT provides a Devaluation Sensitivity Index (DSI), a measure of the balance between relative goal-directed and habitual learning. Individuals with Obsessive-Compulsive Disorder (OCD) often engage in repetitive actions, suggesting a potential deficit in goal-directed control and an increased reliance on habitual learning. Previous literature has shown that medicated OCD adults performed worse on the SOAT task than healthy controls.

**Objectives:**

To compare habit learning performance in an unmedicated sample:

- **Goal 1:** Between OCD and Healthy Controls (HC)
- **Goal 2:** Across four groups: adult OCD, adult HC, children OCD, and children HC

**Methods:**

**Participants:** Eighty-three participants (44 OCD patients and 38 healthy controls) completed the study with usable task data. The 44 OCD patients comprised 17 adults (mean age: 26.76 years, SD: 8.61 years) and 27 children/adolescents (mean age: 12.84 years, SD: 2.59 years). The 38 healthy controls included 17 adults (mean age: 30 years, SD: 7.49 years) and 21 children/adolescents (mean age: 14.1 years, SD: 2.19 years). All participants were unmedicated. **Measures:** Participants completed an adapted version of the “Fabulous Fruit Game”, which included an instrumental training phase to learn Stimulus-Response-Outcomes (S-R-O) associations and a SOAT to assess the strength of learned S-R-O associations. DSI was calculated by subtracting the percentage of responses made toward devalued outcomes from the percentage of responses made toward still valuable outcomes. **Behavioral Analyses:** Student’s t-test comparing individuals with OCD to HC and a ONEWAY ANOVA to examine group differences across multiple categories.

**Results:**

**Goal 1:** DSI comparison between individuals with OCD and HC revealed a significant difference, with HC demonstrating superior performance (t (60.9) = 2.60, p = .012, Cohen’s d = .546). **Goal 2:** The overall DSI comparison across adult OCD, adult HC, children OCD, and children HC showed a non-significant difference (F(3) = 3.407, p = 0.22). However, post hoc analysis revealed significant differences between Adult HC and Youth OCD (I-J Scheffe = 28.82, p = .033), indicating superior performance in adult HC.

**Conclusions:**

This study highlights altered Habit Learning in unmedicated OCD individuals, supported by significant DSI differences compared to HC. Age-related distinctions were observed, emphasizing the need for age-sensitive interventions in understanding and addressing habit-related challenges in OCD.

**Disclosure of Interest:**

None Declared

